# Flora of Nam Kading National Protected Area I: a new species of yellow-flowered *Strobilanthes* (Acanthaceae), *S. namkadingensis*

**DOI:** 10.3897/phytokeys.81.13203

**Published:** 2017-06-08

**Authors:** Phetlasy Souladeth, Shuichiro Tagane, Meng Zhang, Norikazu Okabe, Tetsukazu Yahara

**Affiliations:** 1 Faculty of Forest Science, National University of Laos, Dongdok campus, P.O.Box 7322, Vientiane, Lao PDR; 2 Center for Asian Conservation Ecology, Kyushu University, 744 Motooka, Fukuoka, 819-0395, Japan; 3 Graduate school of systems life sciences, Kyushu University, 744 Motooka, Fukuoka, 819-0395, Japan

**Keywords:** DNA barcoding, Indochina, Laos, *Sericocalyx*, taxonomy

## Abstract

A new species of Acanthaceae, *Strobilanthes namkadingensis* Soulad. & Tagane from Nam Kading National Protected Area, Bolikhamxay Province, central Laos, is described and illustrated. It is characterized by long spicate inflorescences consisting of 6-32 flowers, yellow corolla, the absence of long white hairs on the bracts and 4–6 seeds per capsule. Three DNA barcode regions of the partial genes for the large sub-unit ribulose-1,5-bisphosphate carboxylase oxygenase (*rbcL*) and maturase K (*matK*) and internal transcribed spacers (ITS) are also provided.

## Introduction

*Strobilanthes* Blume, consisting of ca. 400 species is one of the largest genera in the family Acanthaceae (Hu et al. 2011). The genus is characterized by plietesial flowering pattern, homomorphic calyx lobes (sometimes partially fused to form a bipartite or tripartite calyx), 4 monadelphous stamens in which usually 2 filaments are distinctly longer than the other 2, 2-locular ovary with 2(-8) ovules per locule, and bifurcurate stigma with unequal branches (Hu et al. 2011). The species of *Strobilanthes* are widely distributed from lowlands to high mountains in subtropical to tropical areas in Asia. In Laos, 14 species with one subspecies have been recorded (Benoist 1935, Deng et al. 2007, Newman et al. 2007, Wood and Scotland 2009).

Here, we describe a new species of *Strobilanthes* from Nam Kading National Protected Area, Bolikhamxay Province, central Laos. The national park covers an area of 169 ha, with an elevation gradient from 138 m in the lowland to 1,514 m at the summit of Phou Pa Paek and is bisected by the Nam Kading River. The climate is most strongly influenced by the south-west monsoon from April to October that brings 90 percent of the annual precipitation. Temperature in the lowland of Bolikhamxay Province varies between 20°C and 30°C, but in the high altitude areas of Nam Kading Protected Area it may drop to as low as 5°C during dry season from December to February (Hallam and Hedemark 2013). The vegetation of Nam Kading Protected area contains mixed deciduous forest, grasslands, wetlands and limestone karst (Strindberg et al. 2007, Hallam and Hedemark 2013). The only floristic survey of the Area was made near human settlements by Electrowatt (1995) who reported 256 plant species including 2 rare species namely, *Lagerstroemia balansae* Koehne (synonym of *L. cochinchinensis* Pierre ex Laness.) and *Justicia gendarussa* Burm.f.

During a botanical survey in Nam Kading National Protected Area in December 2016, a wild, yellow-flowered species of *Strobilanthes* was collected in the semi-shaded understory of semi-evergreen forest. To determine its identity, we made a morphological comparison to closely related species based on dried specimens at herbarium (BKF, FOF, FU, RUPP and SAR), digital images of specimens on the webpages of JSTOR Global Plants [https://plants.jstor.org/ (accessed 22 Feb. 2017)], K [http://apps.kew.org/herbcat/navigator.do (accessed 22 Feb. 2017)] and P [https://science.mnhn.fr/institution/mnhn/collection/p/item/search (accessed 22 Feb. 2017)], and the relevant literature of surrounding countries including Cambodia, China, Japan, Laos, Thailand and Vietnam (e.g. Benoist 1935, Deng et al. 2007, Newman et al. 2007, Wood and Scotland 2009, Hu et al. 2011).

The purpose of this paper is to describe and illustrate this as a new species, *Strobilanthes namkadingensis* Soulad. & Tagane accompanying with DNA barcodes of the three DNA barcode regions, the partial genes for the large subunit ribulose-1,5-bisphosphate carboxylase oxygenase (*rbcL*) and maturase K (*matK*) (CBOL Plant Working Group 2009) and the internal transcribed spacer region of the nuclear ribosomal DNA (ITS). DNA sequencing for *rbcL* and *matK* followed to the published protocols (Kress et al. 2009, Dunning and Savolainen 2010) and for ITS according to Rohwer et al. (2009) and Chen et al. (2010) using the two primer pairs (ITS18-F, ITS26-R and, ITS2-S2F and ITS2- S3R).

## Taxonomy

### Strobilanthes namkadingensis

Taxon classificationPlantaeLamialesAcanthaceae

Soulad. & Tagane sp. nov.

urn:lsid:ipni.org:names:60474713-2

Figs 1 , 2

#### Diagnosis.

*Strobilanthes namkadingensis* is distinguished from all the previously known species of Laos and its surrounding countries including China, Cambodia, Thailand and Vietnam clearly by a combination of 6–32 flowered spikes up to 10.5 cm long, yellow corolla, the absence of long white hairs on the bracts and 4–6-seeded capsule. In the region, *S. namkadingensis* is similar to *Strobilanthes squalens* S.Moore of Vietnam and *Sericocalyx thailandicus* Bremek. of Thailand in having yellow corolla and long-beaked floral bracts, but distinguished by its long spikes (vs. less than 3 cm long), broader floral bracts (obovate-elliptic to broadly elliptic vs. lanceolate to ovate-lanceolate), smaller corolla (1.9–2.1 cm long vs. less than 1.7 cm long), and the absence of long white hairs on the bracts.

**Figure 1.**
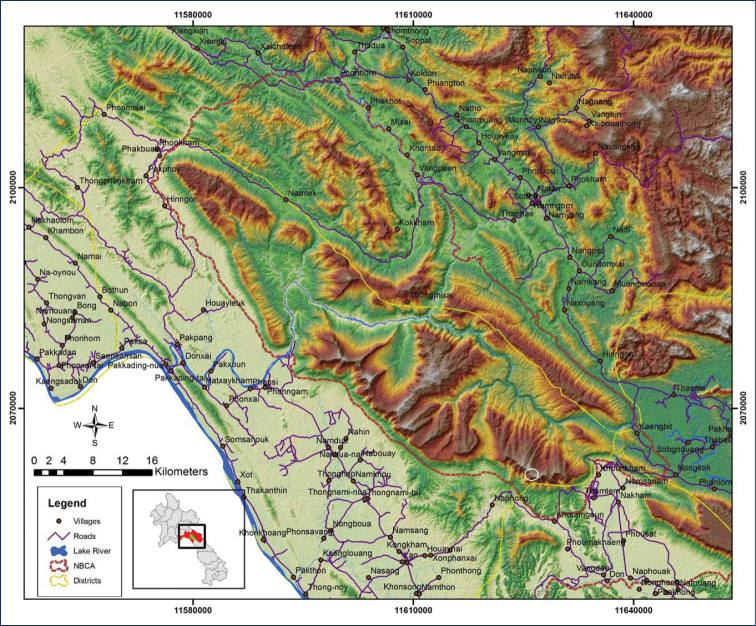
Locality of Nam Kading National Protected Area. White circle indicates where we found *Strobilanthes namkadingensis* Soulad. & Tagane.

**Figure 2.**
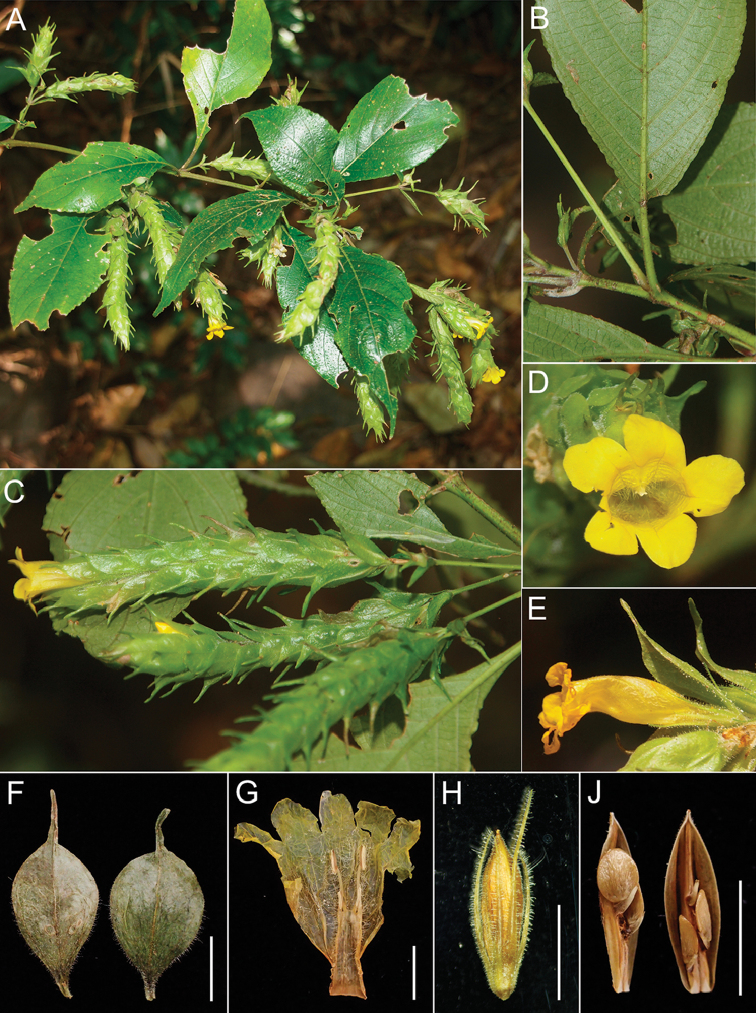
*Strobilanthes namkadingensis* Soulad. & Tagane. **A** flowering branch **B** abaxial leaf surface **C** inflorescence **D** flower **E** side view of corolla **F** floral bracts **G** corolla opened out **H** fruit with calyx **J** longitudinal section of capsule showing seeds (from *Tagane et al. L426*, FU). All scale bars: 5 mm.

#### Type.

LAOS. Bolikhamxay Province, Nam Kading National Protected Area, in semi-evergreen forest, beside a dried rocky stream, 18°12'17.9"N, 104°33'34.5"E, alt. 146 m, 26 Dec. 2016, with flowers and fruits, *Tagane S., Yahara T., Zhang M., Okabe N., Souladeth P., Sengthong A., Chayer S. L426* (holotype-HNL!, isotypes-FOF!, FU!, K, KYO!, P).

#### Description.

Anisophyllous shrub, 1.5 m tall. Stem terete, densely covered with white hairs, pale yellow green when dried. Leaves slightly unequal in each pair, petiolate; blade broadly elliptic, ovate-elliptic, (0.7–)5.5–11 × (0.2–)2.2–4.5 cm, apex acuminate, base cuneate, briefly decurrent onto petiole, margin shallowly crenate, chartaceous, pale yellow green adaxially, light pale yellow green abaxially, sparsely strigose and scabrous on both sides, densely covered with cystoliths adaxially; midrib prominent on both surfaces, secondary veins 6–8 pairs, prominent on both surfaces, tertiary veins scalariform-reticulate, faintly visible to invisible; petiole 0.5–1.3 cm long, scabrid adaxially, glabrous abaxially, margin ciliate. Inflorescences spicate, terminal and axillary, (2–)3–10.5 cm long, 6–32-flowered; outer (inflorescence) bract narrowly elliptic-ovate, 1.4–2.1 × 0.4–0.5 cm, apex acute to acuminate, sessile, shortly puberulent adaxially, sparsely with short stiff hairs at margins and on midveins on both surfaces; floral bracts obovate-elliptic to broadly elliptic, ca. 1.7 × 0.7 cm long, apex caudate, acumen up to 0.8 cm long, persistent, pale yellow green, sparsely pilose with glandular hairs adaxially, shortly puberulent, with grand-tipped hairs abaxially, margin ciliate with large-celled white hairs; bracteole 2, linear, 5–7 mm long, glandular hairy. Calyx lobes 5, linear, ca. 5–9 mm long, apex acute, slightly accrescent in fruit, upper three and lower two calyx lobes are fused ca. 2 mm from the base, covered with cystoliths adaxially, glandular hairy abaxially. Corolla yellow, funnel-shaped, 1.9–2.1 cm long, 0.8 cm in diam., membranaceous, hairy with short erect hairs outside, pilose with long white hairs inside of tube, corolla lobes 5, elliptic to suborbicular, ca. 3.8 × 3.1–3.8 mm, apex rounded. Stamens 4, didynamous; the shorter pairs of filaments ca. 1.5 mm long, with a few white hairs; the longer ones 5–6.5 mm long, pilose with long white hairs except upper 2/5, base of filaments adnate to corolla tube; anthers ca. 2–2.2 mm long, dorsifixed, attached lower 1/3 part of anther (monadelphous). Style 1.2 cm long, sparsely pilose, stigma ca. 2 mm long, glabrous. Capsule narrowly ellipsoidal, 8.4–10 mm long, ca. 2.8 mm in diam., glandular hairy, 4–6-seeded. Seeds spirally arranged on free-central placenta, suborbicular, 2 mm height, 1.8 mm width, 0.2 mm thick, strongly flattened, light yellow brown, glabrous.

#### Distribution.

Laos, Bolikhamxay Province (so far known only from Nam Kading National Protected Area).

#### Habitat and ecology.

*Strobilanthes namkadingensis* is found in semi-shaded understory of semi-evergreen forest beside a dried stream; at alt. 146 m. The flowering and fruiting specimen was collected in December.

#### GenBank accession no.

*Tagane et al. L426*: LC257983 (*rbcL*), LC257984 (*matK*) and LC257953 (ITS).

#### Etymology.

This specific epithet *namkadingensis* refers to the type locality.

#### Primary conservation assessment.

Data Deficient (DD) (IUCN 2012). Only one individual was found along a dried rocky stream beside road. The individual grows in a protected area of Nam Kading National Protected Areas, but the habitat is close to a road and can be easily affected by human disturbance from the road. Further intensive field surveys are needed to evaluate its conservation status.

#### Note.

Bremekamp (1944) treated the species having a yellow corolla and 4–8 seeded capsule as *Sericocalyx* Bremek. and the new species belongs to this group. However, Moylan et al. (2004) demonstrated that *Sericocalyx* is polyphyletic in the subtribe Strobilanthinae, based on phylogenetic analyses using ITS and *trnL-F* sequences and morphology. Therefore a single monophyletic *Strobilanthes* *s.l.* is accepted (Moylan et al. 2004, Deng et al. 2006) and the new species is here described as a species of *Strobilanthes*. The BLAST similarity search based on the ITS sequence of *S. namkadingensis* resulted in homology as high as 543/562 bp (including 17 gaps) and 532/562 bp (including 19 gaps) with the sequence of *S. chinensis* (Nees) J.R.I.Wood & Y.F.Deng (synonym, *Sericocalyx chinensis* (Nees) Bremek.) (GenBank accession no. AY489384) and *Sericocalyx crispus* (L.) Bremek. (AY489383) respectively in the DNA database, supporting that the new species is genetically closely related to the *Sericocalyx* group. *Strobilanthes namkadingensis* is distinguished from *S. chinensis* by having more secondary veins of lamina (6–8 pairs vs. 5 pairs), longer spikes (3.5–10 cm long as opposed to *S. chinensis* up to 3 cm long), and 4–6 seeds per capsule (vs. 8-seeds).

## Supplementary Material

XML Treatment for Strobilanthes namkadingensis
